# Yeast longevity promoted by reversing aging-associated decline in heavy isotope content

**DOI:** 10.1038/npjamd.2016.4

**Published:** 2016-02-18

**Authors:** Xiyan Li, Michael P Snyder

**Affiliations:** 1 Department of Genetics, Stanford University School of Medicine, Stanford, CA, USA

## Abstract

Dysregulation of metabolism develops with organismal aging. Both genetic and environmental manipulations promote longevity by effectively diverting various metabolic processes against aging. How these processes converge on the metabolome is not clear. Here we report that the heavy isotopic forms of common elements, a universal feature of metabolites, decline in yeast cells undergoing chronological aging. Supplementation of deuterium, a heavy hydrogen isotope, through heavy water (D_2_O) uptake extends yeast chronological lifespan (CLS) by up to 85% with minimal effects on growth. The CLS extension by D_2_O bypasses several known genetic regulators, but is abrogated by calorie restriction and mitochondrial deficiency. Heavy water substantially suppresses endogenous generation of reactive oxygen species (ROS) and slows the pace of metabolic consumption and disposal. Protection from aging by heavy isotopes might result from kinetic modulation of biochemical reactions. Altogether, our findings reveal a novel perspective of aging and new means for promoting longevity.

## Introduction

Normal aging is accompanied by progressive metabolism deterioration.^1^ Understanding the basic mechanisms involved in aging and how the process can be manipulated will provide useful insights and treatment for this universal process. Currently, adjusting and diverting metabolic flows serve as a major objective to delay aging. Genetic manipulation of a range of metabolic regulators are implicated in lifespan extension in experimental organisms.^1^ In budding yeast *S. cerevisiae*, these include suppression of anabolism through mTOR inhibition,^2^ activation of catabolism through AMPK^3^ and sirtuins activation,^4^ and enhancement of bioenergetics through mitochondria.^5^ In parallel, calorie restriction (CR, or dietary restriction, DR), as a typical environmental intervention, works equally well as, or in certain cases, even better than genetic manipulation to extend lifespan.^6^ Modulation of metabolism through nutrient uptake may thus represent a deserving avenue of anti-aging intervention.

Two discrete types of aging could be recognized in the budding yeast:^7^ (1) replicative aging which is marked by a gradual loss of dividing capability in single cells; and (2) chronological aging which is defined by diminished survival of nondividing cells. Both aging processes may be retarded by limiting nutrients, including carbon sources (through CR)^4,8^ and amino acids (AAs; through DR),^2,6^ pinpointing the importance of nutrient availability and metabolism in regulating lifespan. Examining the metabolome of yeast cells may provide direct clues to underlying mechanisms involved in aging.

## Results

### The yeast metabolomic decline during aging

**Figure 1:**
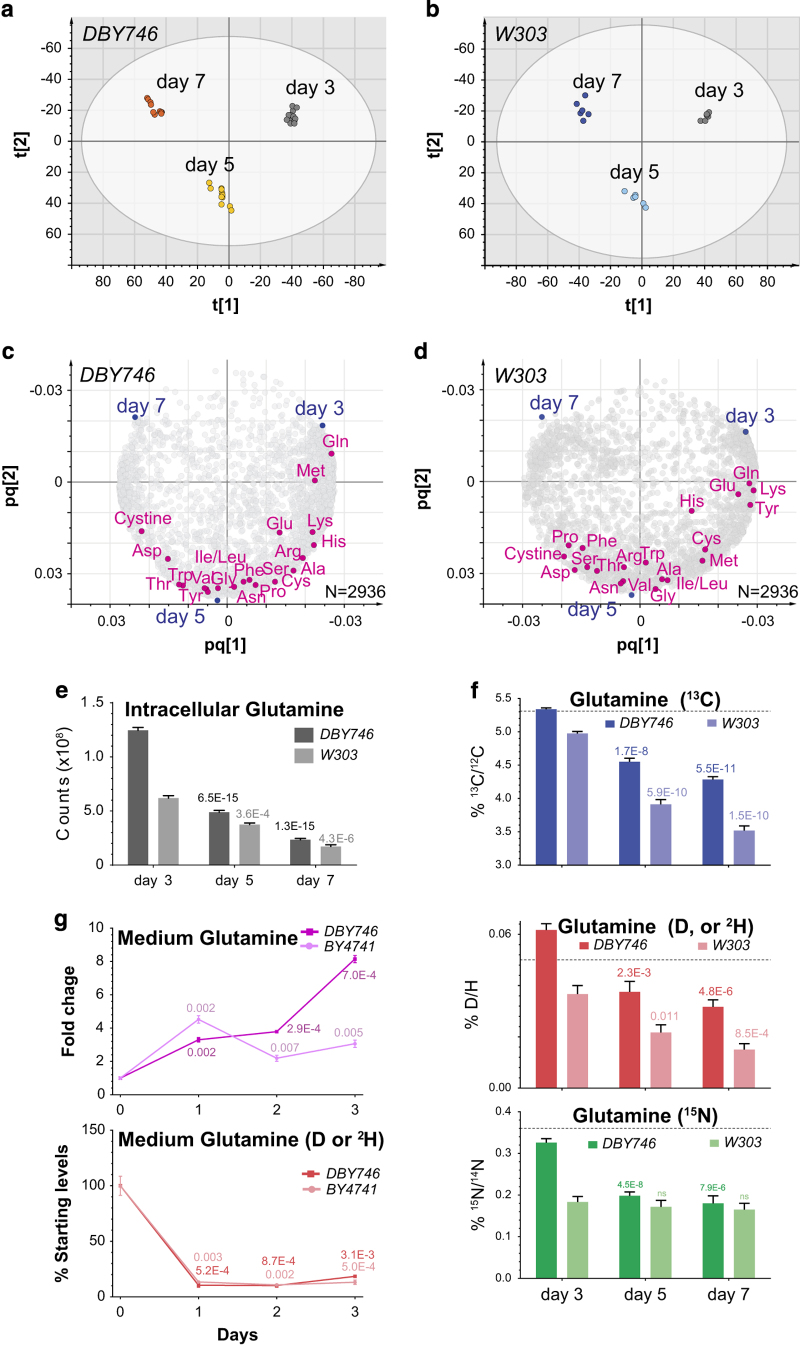
Aging-associated decline in heavy isotopic metabolites in yeast revealed by LC-MS-based metabolomics. See also Supplementary Figure S1, Supplementary Table S2. (**a**, **b**), The intracellular metabolome from yeast cells was examined by LC-MS when cells have reached stationary phase at day 3, 5, and 7 after inoculation (*n*=6 for each time point). The O2PLS-DA clustering for two strains (DBY746, W303) is shown for DBY746 in **a** (R2Y=0.983, Q2=0.979), and for W303 in **b** (R2Y=0.985, Q2=0.955). *X* and *Y* axis are reversed for clarity of comparison. (**c**, **d**) Contribution of individual metabolites to the clustering models in **a**, **b**, respectively. Each circle represents a metabolite (mass feature). All twenty proteinogenic amino acids and cysteine are highlighted in magenta. Our method does not distinguish isoleucine and leucine. (**e**) The plot of the intracellular levels of glutamine (Gln) in **c**, **d**. The *P* values of two-tailed unequal variance *t*-test are shown above each bar for comparison with levels at day 3 for each strain. Error bars=s.e.m. The same type *t*-test was applied in all other analyses in this manuscript unless otherwise noted. (**f**) The relative monoisotopic abundances of glutamine that contains one ^13^C, ^2^H (D) or ^15^N, respectively, normalized to the most abundant mass species composed of all light isotopes (*n*=6). Values were manually calculated in Thermo XCalibur. The *t*-test *P* values are shown above each bar for comparison with levels at day 3 for each strain. Dotted lines indicate the relative monoisotopic abundances of medium glutamine. Error bars=s.e.m. NS, not significant. (**g**) The levels of glutamine and its D-isotopic form in the culture medium in the first 3 days after inoculation for two strains (DBY746, BY4741, *n*=3 each). The *t*-test *P* values are shown above each bar for comparison with starting medium levels. Error bars =s.e.m.

To reveal age-associated changes of metabolome, we first examined the metabolome from yeast cells. For this study, we focused primarily on chronological aging which can be analyzed by standard assays.^9^ In this assay, yeast cells are grown to stationary phase, incubated for varying degrees of time, and the percentage of viable cells determined. We examined yeast cells undergoing chronological aging at 3 time points, using an untargeted liquid chromatography-coupled mass spectrometry (LC-MS)-based metabolomics approach (Figure 1a–d). At day 7, the survival ratio drops to around 50% of the level at day 3 for both DBY746 and W303,^9^ two strains commonly used in chronological aging studies. A total of 2,936 high-confidence metabolites exhibited distinctive clustering with respect to each time point for both strains(R2Y>0.98, Q2> 0.95), indicating a clear trend in age-associated metabolome changes (Figure 1a,b). The contribution of individual metabolites to the multivariate regression clustering model (O2PLS-DA) was correlated with its distance to the placeholder for each time point group (Figure 1c,d). Of 237 metabolites with a KEGG entry, 58 metabolites that showed a decreasing trend with time were shared in both strains, and over-represents a few metabolic pathways, including arginine and proline metabolism (urea cycle) and oxidative phosphorylation (Supplementary Table S2), two processes for energy generation and waste disposal in catabolism that also decline in aging.^10,11^ In contrast, 17 metabolites with an increasing trend were found in both strains and showed no enrichment for any metabolic pathways (Supplementary Table S2). These observed changes in the metabolome over chronological aging corroborates with the decline in general metabolism in other experimental organisms.^1^

In particular, the intracellular levels of 16 free AAs started to decline as late as day 5, reflecting a natural depletion of nutrients in aging population (Figure 1c,d), which also agrees with previous observations that branched-chain AAs decline with replicative aging.^12,13^ In both strains, glutamine (Gln) showed the fastest depletion, as judged by its closest proximity to the day 3 placeholder (Figure 1d and Supplementary Figure S1a).

### The metabolite isotope content decline during aging

Aside from measuring the levels of metabolites, we also examined the isotopic content of metabolites which has not been analyzed previously. Heavy stable isotopes represent a natural small portion of common elements found in biological systems, but may markedlly affect the kinetics of biochemical reactions.^14^ When operated with high mass resolution (resolving power at 100,000 in this study), mass spectrometry is able to distinguish the subtle mass differences between molecular forms that contain different isotopes for the same element, including most common forms such as ^13^C, ^15^N and ^2^H (or D; see Supplementary Figure S2b for an example). Current technical capacity of mass spectrometry can only achieve sufficient separation of isotopic species for a given metabolite with a molecular mass below ~300 Da and with an abundance that expands over three orders of magnitude in MS counts. Consequently, we only manually measured the heavy isotope-containing forms of small metabolites, and report the results for 20 AA because they represent an intracellular metabolite group that is essential for protein synthesis and subject to stringent surveillance at the metabolic level.^15^

Strikingly, the relative abundance of heavy isotope-containing metabolites exhibited an aging-associated trend as well. Whereas less abundant AAs showed an increase in heavy isotope content, more abundant AAs declined in heavy isotope content, suggesting an aging-associated decline in the metabolome (Supplementary Figure S1c). The most prominent AA showing a declining trend was glutamine. Both ^13^C and ^2^H-containing forms of glutamine declined to 50% at day 7 from day 3 as a portion of total glutamine in both strains (Figure 1f). In contrast, glutamate (Glu), a closely related AA, exhibited no obvious trends in relative abundance of all three isotopes despite similar decline in total Glu in the same period of time (Supplementary Figure S1d,e). The aging-associated Gln decline is unlikely due to the lack of availability, as the Gln levels actually increased by 3 to 8-fold in the medium at day 3, as determined by LC-MS (Figure 1g). Conversely, medium Glu levels were rapidly depleted at the same time (Supplementary Figure S1f). The accumulation of Gln in medium likely results from deregulated metabolic waste disposal such as detoxification of ammonia.^16^ Given the prevalence and central importance of Gln in microbial metabolism,^16,17^ the aging-associated decline in the total and isotopic Gln may be a consequence of changes in multiple metabolic processes, including energy generation (e.g., oxidative phosphorylation) and waste disposal (e.g., catabolic amino-acid metabolism). We note that due to technical limitation and vast dynamic ranges of intracellular metabolite content during aging, not all AAs were suitable for this type of thorough measurement, as denoted by the gray color in Supplementary Figure S1c. Nevertheless, this finding raised the novel hypothesis that yeast cells may gradually lose the ability to retain heavy metabolites in aging.

### Yeast chronological lifespan extension by heavy water uptake

**Figure 2:**
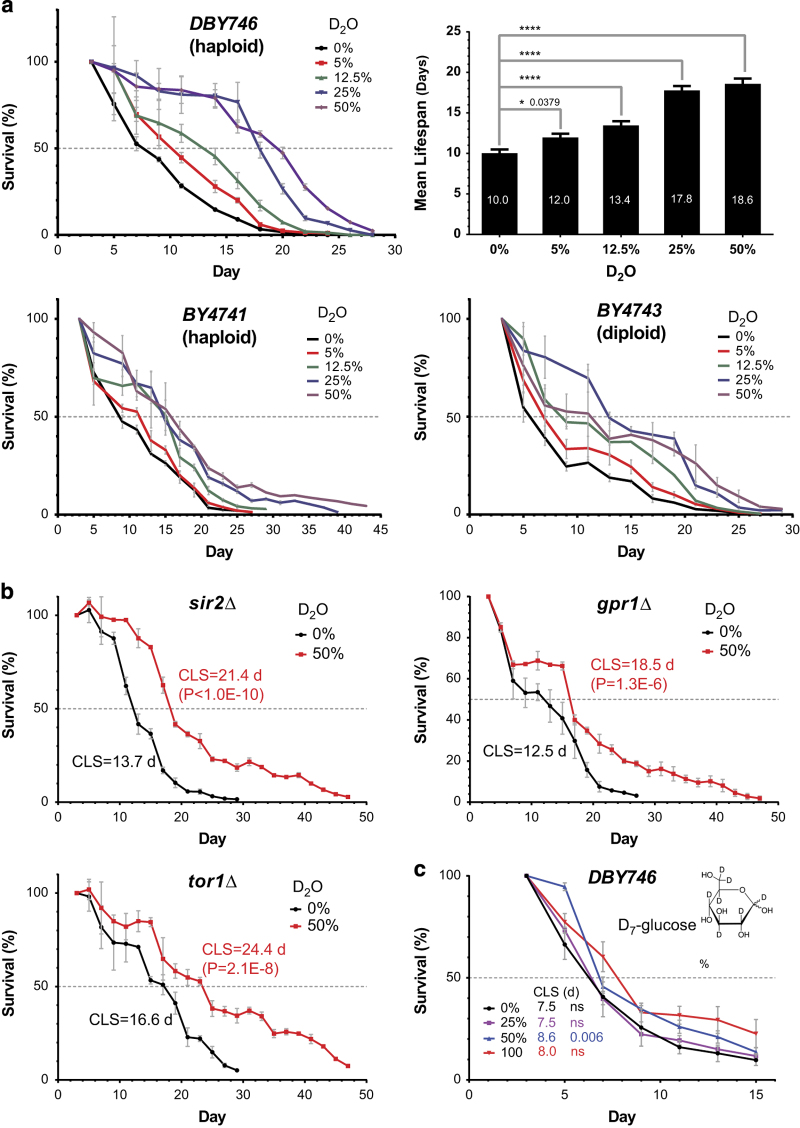
Chronological lifespan extension in yeast by metabolic introduction of heavy hydrogen isotopes deuterium through heavy water (D_2_O). See also Supplementary Figure S2. (**a**) Chronological survival curves for yeast (*S. cerevisiae*) strains DBY746, BY4741 and BY4743, respectively (*n*=3). Chronological aging assays were performed in fortified SDC medium as described previously (see Materials and Methods) for several common laboratory strains. Fresh culture was used to inoculate SDC medium containing different amounts of D_2_O (v/v). Colony formation was determined from day 3 and every 2 days thereafter until the survival rate dropped below 10% (representative images in Supplementary Figure S2A). At least three independent experiments were performed, and one is shown here. Top right panel shows mean lifespans (estimated by Kaplan–Meier survival analysis, shown inside each bar) of DBY746 strain in media containing D_2_O. *P* values (log-rank test after Bonferroni correction) of pair-wise comparisons are indicated above the bars (*, <0.05; ****, <1E-4). Error bars=s.e.m. (**b**) CLS assays of three yeast mutants (BY4741 background) treated with 50% D_2_O, each with a deletion of a known aging regulator gene in yeast (*n*=3). Two independent experiments were performed, and one is shown here. The mean lifespans and *P* values are estimated by log-rank test and indicated on each panel. The experiments were from the same culture presented in **a**. Error bars=s.e.m. (**c**) Deuterium introduced through carbon sources (glucose) failed to extend CLS in yeast to a significant extent. The total glucose concentration is 2% (w/v) in all conditions. D_7_-glucose contains deuterium at all seven of nonexchangeable hydrogen positions (structure shown on right). The mean lifespans and *P* values (log-rank test, versus 0% D_7_-glucose) are indicated with the same color of survival curves. NS, not significant. Error bars=s.e.m.

For many abundant metabolites heavy isotope contents decrease during aging. We therefore tested whether the addition of heavy isotopes might extend chronological lifespan in yeast. We introduced heavy isotope nutrients through medium uptake into haploid and diploid yeast, and determined the mean chronological lifespan by quantifying the time required for surviving colony forming units in a stationary phase culture to drop to 50% (Supplementary Figure S2a), as previously reported.^9^ Feeding yeast with ^2^H (deuterium) through glucose was able to extend the chronological lifespan (CLS) significantly, but not to substantial extent (Figure 2c). This is likely due to the fact that the metabolic incorporation of deuterium through glucose only represents a small portion of hydrogen flux metabolism, therefore it only marginally affects cell metabolism and thus cell aging. We also reasoned that ^13^C incorporation through single nutrient uptake may also fail to effectively affect carbon flux metabolism for similar reasons, as supported by yeast growth experiments (not shown) and noted in parallel stable isotope labeling experiments.^18^

We then tested whether deuterium supplementation through alternative means extends yeast CLS. Because of the limited availability of deuterated AAs and the observation that medium Gln accumulation accompanied intracellular Gln declines during aging (Figure 1e,g), we used heavy water (D_2_O) as the vector to introduce deuterium into the metabolites. Heavy water directly suppresses chemical reactions that involve water splitting or forming, which make up 39% all biochemical reactions in budding yeast.^19^ We observed substantial CLS extension in different yeast strains incubated with increasing doses of D_2_O. D_2_O extended the CLS in a dose-dependent manner in the two haploid and one diploid strains tested, and the maximal CLS extension, upon 50% D_2_O treatment, was 85% (*P*=0.0001) for DBY746 (haploid, a strain commonly used for CLS studies^9^), 50% (*P*=4.1E−8) and 59% (*P*=2.5E−7) for BY4741 (haploid, a strain used for the Yeast Genome Deletion Project), and BY4743 (a diploid strain related to BY4741), respectively (Figure 2b, also see Supplementary Table S1). Importantly, CLS extension was also observed when D_2_O was administrated after the yeast cells reached stationary phase (Supplementary Figure S2f), demonstrating that D_2_O uptake promotes longevity in both proliferative and nonproliferative growth stages.

The CLS extension is unlikely due to any potential influence of D_2_O on cell growth, as the initial colony formation capacity or cell volumes at the beginning of the lifespan assays were not substantially altered by D_2_O (Supplementary Figure S2b,c). Besides, according to previous study, D_2_O only had negligible effect on oxygen consumption in the dosage range used in this study.^20^ Furthermore, growth curve assays showed D_2_O only slightly affected the culture at the log phases (Supplementary Figure S2d,e), which substantially precedes when all cultures reached saturation phase at day 3, the first time point for all CLS assays. The growth capability of yeast cells did not appear to be affected even after prolonged exposure (40 days) to D_2_O (Supplementary Figure S2g,h), suggesting that the observed lifespan extension is unlikely the consequence of a biased selection for spontaneous mutations promoting fitness, which could skew the assay.

### Bypassing of genetic regulators by D_2_O in CLS extension

To position the heavy isotope effects in relation to genetic CLS regulation, we tested whether D_2_O extends yeast CLS in the absence of known aging regulators (BY4741 background). We determined the mean CLS in yeast strains with gene deletions in *GPR1*, *SIR2* or *TOR1*, each of which has a reported role in affecting yeast lifespan.^4,9,13,21,22^ Consistent with previous studies, we confirmed that strains with a deficiency in *TOR1* are long-lived (44.9%, *P* value 3.9E−8) (Figure 2b). The mean CLS of each mutant strain was extended by D_2_O by a level comparable to that in the wild-type strain (*gpr1Δ*, 48.3%; *sir2Δ*, 56.5%; *tor1Δ*, 47.0%, versus 50% in BY4741) (Figure 2b). These results showed that D_2_O bypasses these genes in its capability to extend CLS, and thus D_2_O likely acts on downstream or in parallel to these genetic regulations in promoting longevity, which also corroborates its minimal effects on yeast growth (Supplementary Figure S2b,e).

### CLS extension by D_2_O mediated by metabolism

**Figure 3:**
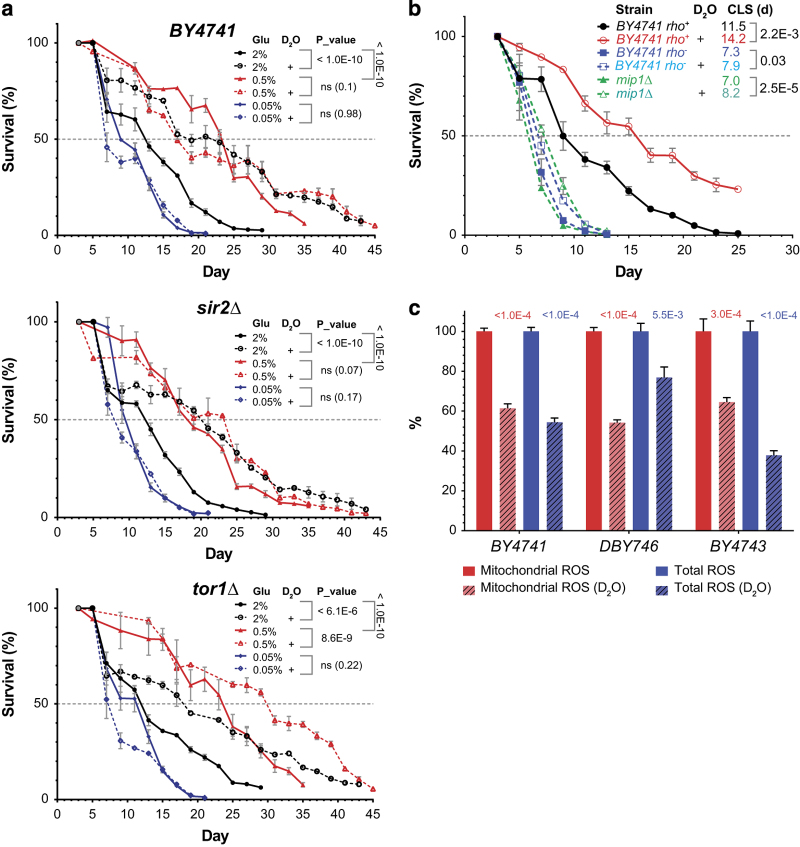
Heavy water extension of yeast chronological lifespan mediated by calorie restriction and mitochondria. See also Supplementary Figure S3. (**a**) CLS extension by D_2_O (50%) is abrogated by mild calorie restriction (*n*=3). Two independent experiments were performed, and one is shown here. The *P* values (log-rank test) for comparison with respective control (no D_2_O) are indicated in the legends. The *P* values (log-rank test) for comparison with 2% glucose are<1.0E−5 for all other five conditions. NS, not significant. Error bars=s.e.m. (**b**) CLS extension by D_2_O (50%) is attenuated in yeast cells lacking mitochondria. Mitochondria-deficient petite strains (*rho* ^−^) were generated through EtBr treatment (see Materials and Methods). The deletion strain *mip1Δ* (BY4741 background) lacks the gene encoding the single subunit of the mitochondrial DNA polymerase in *S. cerevisiae*. Lifespans upon treatment with 50% D2O is shown. The mean lifespans and *P* values (log-rank test, versus BY4741 (*rho* ^*+*^, 0%) are indicated with colors respective to legends. *n*=3. Error bars=s.e.m. (**c**) Measurement of *in vivo* generation of reactive oxygen species (ROS). The ROS generation was continuously monitored by fluorescent dyes CellROX (total ROS) and MitoSOX (mitochondrial ROS) on a plate reader for three strains treated with D_2_O (50%) (*n*=3). The ROS generation, as calculated by area under curve calculation in Supplementary Figure S3, was normalized to no D_2_O controls for each strain (control as 100%). The *P* values (two-tailed *t*-test, unequal variance) are indicated on graph. Error bars=s.e.m.

Heavy isotopes greatly affect the kinetics of biochemical reactions, the chemical basis for metabolism *en masse*. The metabolic decline in aging may lead to decline in heavy isotope content in the metabolome (Figure 1). Conversely, because D_2_O suppresses all hydrolysis reactions, which are mostly catabolic, D_2_O treatment may retard aging-related metabolic deterioration. To test whether CLS extension by D_2_O is mediated by metabolism, we applied D_2_O treatment under calorie restriction (CR) conditions in yeast. CR works as an effective environmental intervention to activate catabolism and extend yeast lifespan.^6^ Different CR regimens act through different intracellular pathways in yeast.^4,23,24^ Consistent with previous reports, we observed that mild CR (0.5% glucose) extended the mean CLS in all three genetic conditions (BY4741 wild-type, *sir2Δ* and *tor1Δ*) analyzed, whereas extreme CR (0.05% glucose) showed deleterious effects on CLS (Figure 3a). Under the mild CR condition, D_2_O failed to further extend the prolonged lifespans of either wild-type or *sir2Δ* mutants; yet, it still caused significant lifespan extension in *tor1Δ* mutants (Figure 3a). Both *SIR2* and *TOR1* have reported roles in mediating CR responses in yeast: *SIR2* mainly operates at mild CR by maintaining chromatin integrity in an NAD-dependent manner, whereas *TOR1* works more efficiently under extreme CR by modulating mitochondrial functions.^4,8,9,23,25^ Consistent with these findings, our observations suggest that D_2_O may mimic the mild CR condition to promote chronological longevity in yeast, possibly owing to their abilities to elicit catabolic activation. These findings together tether D_2_O-promoted longevity to calorie restriction and highlight the importance of delicate control of metabolism in yeast aging.

### CLS extension by D_2_O mediated by mitochondria

In eukaryotes, mitochondria serve as the central hub to orchestrate material and energy metabolism, thus are the target of many aging regulators.^1,26^ To test whether mitochondria mediate D_2_O-promoted longevity, we conducted CLS assays on yeast strains with mitochondrial deficiency. The *mip1Δ* strain carries deletion of the gene encoding the sole yeast mitochondrial DNA polymerase gamma subunit^27^ and are short-lived (39.4% reduction in mean CLS versus wild type, *P*<1E−10; Figure 3b). Whereas D_2_O extended the mean CLS of wild-type (BY4741) significantly (*P*=0.0022), it failed to increase the CLS of *mip1Δ* mutants to the same magnitude (Figure 3b). We also generated *petite* (*rho* ^−^) strains through ethidium bromide-induced mutagenesis.^28,29^ Petite strains are unable to grow on nonfermentable medium owing to loss of mitochondrial respiratory functions. Similarly, these petite strains did not show substantial lifespan extension in response to D_2_O treatment (Figure 3b). A modest but statistically significant CLS extension by D_2_O in both mitochondrion-deficient conditions was observed (*P* values in Figure 3b), which could suggest anaerobic metabolism is also involved but is beyond the validity range of our CLS assays. Nevertheless, these results clearly demonstrate the strong dependence of the D_2_O-promoted longevity on mitochondrial functions.

Heavy isotopes have been proposed to potentially confer cells chemical resistance to reactive oxygen species (ROS),^14^ a group of metabolic products that cause chemical damage and are implicated in aging.^4,30,31^ To test whether D_2_O uptake affects endogenous ROS, we continuously monitored the total ROS and mitochondrial ROS generation in D_2_O-treated live cells of three yeast strains using a fluorescence-based method (Supplementary Figure S3). Significant suppression of endogenous ROS generation by D_2_O was observed for both total and mitochondrial ROS, with maximal mitochondrial ROS suppression by 46% (*P*=2.7E−7) in DBY746 and maximal total ROS suppression by 62% (*P*=1.5E−4) in BY4743 (Figure 3c). These results strongly link D_2_O to ROS suppression. It is unknown whether D_2_O suppresses ROS production by reducing free radical damages to intracellular targets, or by decreasing oxidative phosphorylation, a major source of intracellular ROS, or both.^10,31,32^ In summary, we found that D_2_O reduces chronological aging.

### Deuterium incorporation into the metabolome

**Figure 4:**
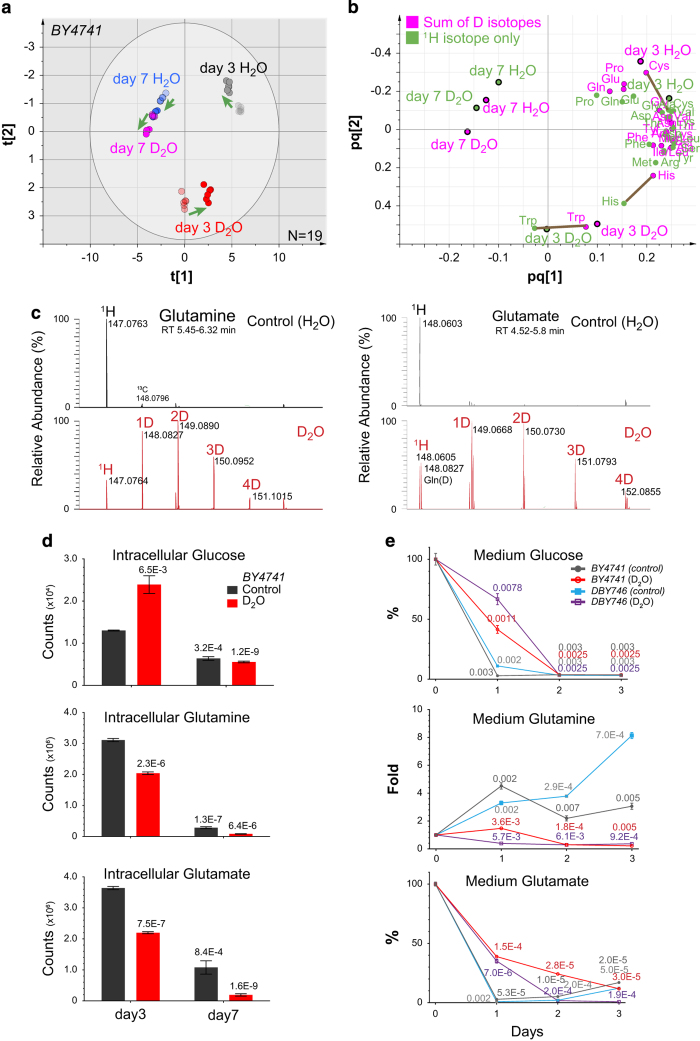
The metabolome shift by incorporation of deuterium through heavy water. See also Supplementary Figure S4. (**a**) The intracellular metabolome of BY4741 yeast cells upon D_2_O treatment at day 3 and day 7 was examined by LC-MS and clustered for all twenty proteinogenic amino acids (see Supplementary Figure S4A for global clustering). Two O2PLS-DA clustering models are superimposed to indicate the metabolome shift in amino-acid (AA) levels caused by D_2_O treatment. Green arrows indicate the direction from the model considering only ^1^H-isotopic AAs (H isotope model, R2Y=0.956, Q2=0.916, half transparent) to the model that sums all deuterium isotopes for each AA (All D-isotope model, R2Y=0.926, Q2=0.87, opaque). Each circle represents one of two technical replicates each from three biological samples. (**b**) The contribution of individual AA to the O2PLS-DA clustering models in **a**. Each circle represents an AA. Brown lines linked three AAs that exhibited minimal deuterium incorporation. (**c**) Extensive deuterium incorporation in two metabolites glutamine and glutamate. Each panel shows one AA and its deuterated derivatives (red trace) in yeast cells (BY4741) grown in medium without D_2_O or with 50% D_2_O at day 3. Accurate masses of relevant mass peaks (*m*/*z*) are indicated on each mass peak. (**d**) Intracellular metabolite levels shifted by D_2_O treatment. The intracellular sums of all hydrogen-isotopic metabolites for glucose, glutamine and glutamate from yeast cells (BY4741) at day 3 and day 7, as summarized in **a**, **b**, are plotted (*n*=3). *P* values for *t*-test versus levels in day 3 control are indicated above each bar. Error bars=s.e.m. (**e**) Medium metabolite consumption pattern reversed by D_2_O treatment. The medium levels of glucose, glutamine and glutamate in the first 3 days of culture after inoculation for two strains (DBY746, BY4741, *n*=3 each). The *t*-test *P* values are shown at each time point for comparison with starting medium levels. The control curves (0% D_2_O) for glutamine (middle panel) is the same as in Figure 1g. Error bars =s.e.m.

To investigate how D_2_O uptakes alters the metabolism, we analyzed the intracellular metabolome of yeast cells undergoing chronological aging upon D_2_O treatment by LC-MS. A total of 4008 metabolites formed discrete primary clusters between day 3 (start) and day 7 (50% survival), which were also separated by D_2_O treatment along the secondary direction (Supplementary Figure S4a). Interestingly, 20 AAs also formed clusters showing a pattern very similar to that of the total metabolites (Figure 4a). To assess how deuterium incorporation may affect the metabolome, we compared two AA clustering models: one for those AAs that considers only the ^1^H-isotopic forms, and another using the sums of all D-isotopic forms for each AA (Figure 4a). Only minimal shift was observed whereas the overall cluster positioning remained almost unaffected (Figure 4a), suggesting that D_2_O did not skew the omic composition of AAs or the entire metabolome.

From the metabolomics perspective, AAs differed greatly in the extent of deuterium incorporation, with extensive incorporation in metabolically active AAs, such as Gln and Glu, but negligible incorporation in AAs that are non-limiting or auxotrophic, such as tryptophan and histidine (Figure 4c and Supplementary Figure S4b,c). Furthermore, extensive deuterium incorporation was not commensurate with metabolome clustering, which is evidenced by the close proximity of each AA between models, considering all ^1^H-isotopic forms and sum of all D-isotopic forms (Figure 4b). Only three AAs were observed with substantial shifts in distance, including tryptophan, histidine and cysteine, all of which had negligible deuterium incorporation (for example, see tryptophan in Supplementary Figure S4b,c). These results implied that at least the other 17 AAs were connected by metabolism involving D_2_O, which as a group showed no compositional response to D_2_O uptake. The dynamics of metabolism may also explain the low toxicity of D_2_O in a range of organisms, including tolerance to pure D_2_O by yeast.^33,34^

To examine how D_2_O affects the metabolic capacity, we analyzed the intracellular and media levels of the same metabolites that are consumed by yeast or disposed of into the media. Surprisingly, the aging-associated decline in intracellular metabolites was not reversed by D_2_O (Figure 4b,d). Instead, D_2_O elicited an intracellular ‘starvation’ of AAs as early as day 3 (Figure 4b), which may mimic the pro-longevity effects by suppression of mTOR, a regulator of AA uptake.^2,25^ Intriguingly, the intracellular glucose was elevated by D_2_O only at early stage (Figure 4d). As the overall growth was not altered by D_2_O (Supplementary Figure S2b,e), the elevation in intracellular glucose at day 3 may result from improved metabolic conservation by D_2_O, which could indicate a modest reduction in glycolysis or a more balanced coupling of glycolysis and its downstream aerobic respiration processes. This observation appears to corroborate the association of increased longevity with reduced insulin and IGF-1 signaling in mammals.^1,35^

D_2_O also alleviated nutrient consumption and metabolic waste disposal, which appears in favor of long-term survival under conditions with limited resources. In early growth stages before day 3, D_2_O uptake not only delayed the decline of medium nutrients, such as glucose and AAs (Glu, Asn, Asp and Thr); it also slowed the accumulation of certain metabolic waste, such as glutamine^16,17^ and hypoxanthine (Figure 4d and Supplementary Figure S4d), both are negatively associated with replicative lifespan.^12,13^ In contrast, the consumption of tryptophan and cysteine was unaffected by D_2_O, coincident with the absence of deuterium incorporation in these metabolites (Figure 4b). These results indicate the metabolic basis for D_2_O uptake as a unique environmental intervention for long-term metabolic conservation and stabilization associated with longevity.

## Discussion

Stable heavy isotopes of common elements in living organisms are prevalent in nature. Although existing in much lower abundance than their light counterparts (1.1% C for ^13^C, 0.1% H for ^2^H or D, and 0.5% N for ^15^N), heavy isotopes produce remarkable kinetic difference because the gain in weight to charge increases the stability of heavy isotope-containing chemical bonds (e.g., by 5- to 10-fold for ^2^H–C over H–C), thus they may substantially slow the reactivity of the chemical bonds and make them less prone to chemical damages—this phenomenon is called kinetic isotope effects (KIE).^14^ The heavy isotopes may not affect biology over short periods because of their natural scarcity, but they could have strong effects after long-term accumulation resulting from massive flux, such as in the discrimination against ^13^C in photosynthesis^36^ and in the metabolism of aging as described in this study (Figure 1). Owing to technical limitation, we only reported aging-associated heavy isotope decline in a group of small metabolites, AAs, but this decline may also exist in other metabolites. One noteworthy group are the lipids as we observed that many lipid species were completely deuterated upon D_2_O treatment (data not shown). Given the prevalent connections and robust dynamics of glutamine in energy and nitrogen metabolism,^16,17^ the heavy isotope decline in glutamine may represent the outcome of overall metabolic fluxes that are well conserved in all eukaryotes.^37^ The heavy isotope decline in yeast during aging may derive from preferential consumption that favors heavy isotopic metabolites or preferential disposal that favors light isotopic metabolites, or both, as suggested in the case of glutamine (Figure 1g). As such we speculate that catabolism may be more sensitive than anabolism in discriminating over isotopic metabolites in the case of D_2_O, which might be related to the fact that water-splitting reactions (13%) are twice as common as water-producing reactions (7%) in yeast and even more in other organisms.^19^ Catabolic stimulation by D_2_O treatment may also explain its overlapping pro-longevity effects with calorie restriction as described in this study, as CR is thought to promote longevity through activation of catabolism.^6^ Altogether, our findings thus reported a natural decline in heavy isotopes in aging yeast, which may be used to assess similar metabolic decline associated with aging in other organisms including humans.

The dependence of D_2_O on mitochondria to extend lifespan highlights a pivotal role of metabolism in aging (Figure 3b). Despite the diversity of genetic adaptation in metabolism as a consequence of organismal evolution and environmental influence, calorie restriction appears to be the only anti-aging intervention that works across all experimental organisms, and perhaps in humans.^1^ By demonstrating that the D_2_O-dependent lifespan extension overlaps with mild CR (Figure 3a) and requires mitochondria (Figure 3b), our results strongly support this notion that metabolic and mitochondrial regulators could regulate the process of aging in a number of organisms.^1,38^ Human population studies have linked higher resting metabolic rates to higher mortalities,^39^ suggesting that slowing the rate of basal metabolism might also retard human aging. It is still unclear how metabolism retardation, at the expense of possible delayed development and reduced survival, could benefit organismal lifespan. Heavy water generally retards growth and metabolism in many multicellular organisms,^40^ but it promoted chronological longevity in budding yeast (this study). Selective removal of mitochondria could also reduce growth in yeast.^25,28,29^ Heavy water has been shown to inhibit H_2_O_2_ production in rodent mitochondria.^41^ Higher levels of reactive oxygen species turnover, perhaps as the consequence of elevated mitochondrial activities, have recently been shown to negatively predict longevity of *C. elegans.* ^30^ Our findings further confirmed that heavy water suppresses endogenous production of cellular and mitochondrial reactive oxygen species (Figure 3c). Altogether, these observations highlight a negative correlation of longevity with mitochondrial malfunctions, including metabolism deregulation, and mitochondria could serve as the major target for D_2_O to elicit prolongevity responses.

Although we do not fully understand the intracellular molecular mechanisms through which heavy isotopes retard aging, we speculate that the mode of actions may result directly from metabolic reactions, such as starvation-like responses and protection from ROS (Figures 3 and 4). We note that our method can only detect deuterium incorporation at the nonexchangeable element positions in each metabolite (Figures 1 and 4), and in the case of hydrogen, this includes C-H bonds but not N–H, O–H or S-H bonds. Therefore, the observed stable incorporation of deuterium predominantly comes through metabolism and is less likely subject to potential contamination from experimental conditions of LC-MS. We showed that D_2_O does not alter the metabolome structure on a global basis with respect to aging (Figure 4), nor does it require known genetic regulators of CR or DR to extend yeast lifespan (Figure 2), suggesting in each case that the overall biological functions are not extensively altered at the genetic levels. Conversely, the dependence of CLS extension by D_2_O on CR and mitochondrial functions (Figure 3) and the failure of CLS extension through ^2^H-glucose feeding (representing lesser KIE) (Figure 2) strongly argue that metabolic actions are critical to longevity promotion by heavy isotopes. Besides, D_2_O slows the pace for both nutrient consumption and waste disposal (Figure 4 and Supplementary Figure S4) but not the growth (Supplementary Figure S2), suggesting that heavy isotopes act bilaterally on the metabolism in favor of metabolic conservation, which may ultimately lead to better long-term sustainability and longevity. Although we only showed evidence for hydrogen isotopes, other elements may also prove to be useful when an effective strategy for delivery is devised. It is also possible that the administration of heavy isotopes might extend lifespan in higher organisms, although it remains unknown whether they might interfere with developmental and biological functions in such organisms. However, it has been shown that the D_2_O doses that extend lifespan do not affect the growth of yeast (Figure 2) or the fertility of fruit flies.^42^

In conclusion, the decline of heavy isotope content in metabolites represents a novel feature of aging and could be a new target to promote longevity by environmental intervention. Specifically, supplementation of heavy isotopes through metabolism could provide a new avenue to control aging and age-associated diseases.

## Materials and methods

### Strains

Strains are listed in Supplementary Table S1. Gene deletion strains were retrieved from the Yeast Genome Deletion Project collection, and all strains were generated in the BY4741 background.

### Media

Yeast SDC media were prepared as previously described^9,43^ with fortified fourfold AAs in surplus, which is auxotrophic to the yeast strains used. YPAD media contained yeast extract (1% w/v), peptone (2%, w/v), dextrose (2% w/v), and adenine hemisulfate (60 mg/l). Heavy water (deuterium oxide, 99.9 atom % D) was purchased from Sigma-Aldrich (St Louis, MO, USA), and was filtered with 0.2-μm PES filter before use. D_7_-glucose (D-Glucose-1,2,3,4,5,6,6–D_7_) was purchased from Sigma-Aldrich (Cat. 552003), and prepared in 40% w/v stock solution before use.

### Metabolomics sample preparation

Yeast culture were centrifuged at 5,000*g* for 5 min at room temperature to collect cells, followed by twice wash in water (MS grade). The cell pellet was flash frozen in dry ice bath until processing. For LC/MS analysis, frozen cell pellet (or medium) of equal cell counts (by absorption at 600 nm) was mixed with dry ice-cool 80% methanol (mass-spec grade) at the same ratio for the same sample batches (usually 1 vol: 10 vol solvent), and then quickly thawed on heat block set at 50 °C for 5 min. The suspension was then processed by three rounds of 1-min vortex at max speed, chilled briefly on ice. The mixture was incubated at 4 °C for 15 min before centrifugation at 20,000*g* for 20 min at 4 °C. The supernatant was stored at −20 °C and used as metabolite extract for LC-MS analysis. For LC-MS analysis, the metabolite extract was transferred to 150-μl deactivated glass insert housed in Waters 2-ml brown MS vials. Chemical standard solution was prepared from 1× synthetic complete mixture from Sigma-Aldrich (Y1501).

### LC/MS acquisition

LC/MS analysis was performed in a platform that consists of Waters UPLC-coupled Exactive Orbitrap Mass Spectrometer (Thermo, Waltham, MA, USA), using a mix-mode OPD2 HP-4B column (4.6×50 mm) with a 4.6×10 mm guard column (Shodex, Showa Denko, Tokyo, Japan). The column temperature was maintained at 45 °C. The sample chamber was maintained at 4 °C. The binary mobile phase solvents were: A, 10 mM NH_4_OAc in 10:90 Acetonitrile:water; B, 10 mM NH_4_OAc in 90:10 Acetonitrile:water. Both solvents were modified with 10 mM HOAc (pH 4.75) for positive mode acquisition, or 10 mM NH_4_OH (pH 7.25) for negative mode. The flow was set as: flow rate, 0.1 ml/min; 0-15 min, 99% A, 15–18 min, 99% to 1% A; 18-24 min, 1% A; 24–25 min, 1% to 99% A; 25–30 min, 99% A. The MS acquisition was in profile mode and performed with an ESI probe, operating with capillary temperature at 275 °C, sheath gas at 40 units, spray voltage at 3.5 kV for positive mode and 3.1 kV for negative mode, Capillary voltage at 30 V, tube lens voltage at 120 V and Skimmer voltage at 20 V. The mass scanning used 100,000 mass resolution, high dynamic range for AGC Target, 500 ms as Maximum Inject Time and 70–1,000 m/z as the scan range.

### LC/MS data analysis

The raw LC/MS data files were centroided with PAVA program^44^ and converted to mzXML format by an in-house R script (distribution upon request). Mass feature extraction was performed with XCMS v1.30.3.^45^ The mass features were then manually searched against the Metlin metabolite database using 5 p.p.m. mass accuracy. Retention time matching with compounds in the standard mixture was also performed for a portion of the metabolite hits. The scored mass features were clustered with SIMCA v13.03 (Umetric, Malmö, Sweden).

### Chronological lifespan assay

The CLS assay was carried out as previously described.^9,43^ Fresh colonies were inoculated into SDC media. Overnight culture was seeded into media containing H_2_O or respective doses of D_2_O, and the cultures were kept on a roller drum at 28 °C. Starting from day 3 and then every other day thereafter, a small amount of the culture was taken out and diluted and plated on YPD plates to determine colony formation after 2 days of incubation at 30 °C. Day 3 is considered as the point of 100% survival. Kaplan–Meier Estimator was used to calculate the mean lifespan, using the online software OASIS,^46^ the *P* values were calculated by Log-Rank Test.

### Metabolic pathway enrichment analysis

The KEGG IDs of those metabolites with high discriminatory power were used for pathway enrichment analysis with IMPaLA.^47^ Manual measurement of monoisotopic metabolites was performed in Thermo Xcalibur v2.1 software, with the capability to simulate monoisotopic peaks containing heavy isotopes.

### Petite clone generation

The petite strains were generated following established protocol.^28,29^ Fresh overnight cultures were diluted 1:10 with YPD medium, and treated with ethidium bromide (10 μg/ml) for 3 h at 28 °C. The culture was then plated on YP plates containing 0.1% glucose and 3% glycerol as carbon source for petite and grande selection. The petite phenotype was confirmed by the failure of treated cells to grow on YP media containing only 3% glycerol.

### Plate-based growth assay

Yeast growth assay was performed in 96-well plate format. Fresh overnight cultures were washed twice in water and inoculated in respective experimental medium. The plate was then incubated in a Tecan plate reader at 30 °C with agitation. Optical density at 600 nm was determined every 15 min during a period of 2 to 3 days. The growth curve parameters were deduced by R package grofit.^48^

### Plate-based assay for reactive oxygen species production *in vivo*

Yeast cell cultures at stationary phase were added to 96-well plate. Two fluorescent dyes, CellROX deep red (total ROS, C10422, Life Technologies, Carlsbad, CA, USA) and MitoSOX (mitochondrial ROS, M36008, Life Technologies), were added separately to final concentrations of 5 μM. Hoechst 33342 (H3570, Life Technologies) was added to 5 μg/ml to monitor the cell density. The cultures were incubated at 30 °C with shaking on a Tecan plate reader. The fluorescence was monitored every 15 min for 25 h with fluorescence settings: CelROX, Ex/Em 640/665 nm; MitoSOX, Ex/Em: 510/580 nm; Hoechst 33342, Ex/Em: 350/461 nm. Band width was 10 nm. Fresh media with respective fluorescence dyes were used as blank controls for autofluorescence.

### Cell volume measurement

Fresh overnight yeast culture was added at a ratio of 1:400 to SDC medium containing different amounts of D_2_O. On day 2, the mean cell volumes were determined with a Coulter counter after a 2,000-fold dilution.
