# Supplementary material for: Yeast longevity promoted by reversing aging-associated decline in heavy isotope content
Source: NPJ Aging Mech Dis. 2016 Feb 18;2:16004–. doi: 10.1038/npjamd.2016.4 (PMC5515009; doi:10.1038/npjamd.2016.4)
Supplement: Supplementary Information [file npjamd20164-s1.doc]

**Supplementary Information**

**Yeast Longevity Promoted by Reversing Aging-associated Decline in Heavy Isotope Content**

Xiyan Li^1^ and Michael P Snyder^1,*^

^1^: Department of Genetics, Stanford University, Stanford, CA 94305-5120

**Supplementary** Table 1. Yeast strains used in this study

| **Strain** | **Genotype** |
| --- | --- |
| BY4741 | *MATa his3Δ1 leu2Δ0 met15Δ0 ura3Δ0* |
| BY4743 | *MATa/α his3Δ1/his3Δ1 leu2Δ0 /leu2Δ0 lys2Δ/LYS2 MET15/met15Δ0 ura3Δ0 /ura3Δ0* |
| DBY746 | *MATα leu2-3,112 his3Δ1 trp1-289 ura3-52 GAL+* |
| W303 | *MATa leu2-3,112 trp1-1 can1-100 ura3-1 ade2-1 his3-11,15* |

**Supplementary Table 2. Metabolic pathway enrichment analysis of chronological aging metabolomics. Overlapping metabolites between DBY746 and W303 (as in Fig. 1, C and D) with decreasing trends (pq1>0 in Fig. 1, C and D, N=58) were selected for metabolic pathway enrichment analysis in IMPaLA. The Q-value cut-off were < 0.05 for significant pathway enrichment. Metabolites are listed by KEGG ID. Overlapping metabolites with increasing trends (pq1<0 in Fig. 1C and D, N=17) have not significant enrichment for any metabolite pathway analysis.**

| **Pathway_Name** | **Overlapping_Metabolites** | **No. Pathway Metabolites** | **P value** | **Q value** |
| --- | --- | --- | --- | --- |
| Arginine and proline metabolism (Urea Cycle) | C00064; C03406; C00019; C01137; C04281; C00624 | 91 | 3.22E-05 | 0.00064 |
| Oxidative phosphorylation | C00004; C00003; C00009 | 16 | 0.000169 | 0.00186 |
| ABC transporters | C00064; C00245; C00009; C00135; C00047; C00089 | 123 | 0.000176 | 0.00193 |
| Protein digestion and absorption | C00064; C00047; C00135 | 47 | 0.00421 | 0.0212 |
| Aminoacyl-tRNA biosynthesis | C00064; C00047; C00135 | 52 | 0.0056 | 0.0269 |

**SUPPLEMENTARY FIGURE LEGENDS**

**Supplementary Figure 1. Isotopic abundance in yeast chronological aging process. Related to Figure 1.**

**a,** An example of LC/MS quantification method used in this study. The glutamine levels in Fig. **1a** is calculated by the sum of spectral counts. The peaks are normalized to the absolute counts of the day 3 sample. X-axis, time of elution; Y-axis, relative abundance by spectral counts.

**b,** Isotopic mass peaks separated by mass spectrometry at 100,000 mass resolution. Discrete isotopic mass peaks for carbon (^13^C), nitrogen (^15^N), and hydrogen (^2^H or D) in glutamine were captured by our LC/MS method. The representative mass spectra of glutamine from a BY4741 culture at day 3, 5, and 7 are shown in orange, green, and blue, respectively, and compared with those of simulation (gray) and chemical standards (black, as in the medium). The hydrogen isotopes are also indicated in the inset by the yellow arrow.

**c,** Trend changes of common heavy isotopes in amino acids from day 3 to day 7 (n=6). MS counts of each AA was normalized to the counts of a standard mixture containing all twenty AAs at defined concentration. Cutoff: P< 0.01 (t-test). ns, not significant.

**d,** Cellular levels of glutamate in the same experiment described in Fig. **1e** (n=6). Error bars = SEM. ns, not significant.

**e,** The relative monoisotopic abundances of glutamate that contains one ^13^C, ^2^H (D), or ^15^N, respectively, to the most abundant mass species composed of all light isotopes (as in Fig. **1f**, n=6). Dotted lines indicate the relative monoisotopic abundances of medium glutamate. Error bars = SEM.

**f,** The medium levels of glutamate in the first 3 days of culture after inoculation for two strains (DBY746, BY4741, n=3 each). The t-test P values are shown above each bar for comparison with starting medium levels. Error bars=SEM.

**Supplementary Figure 2. Yeast chronological lifespan assays. Related to Figure 2.**

**a,** Representative photographs of chronological aging assays demonstrate the changes in colony numbers during chronological aging. The time points into the assay are shown above, and the doses of D_2_O used for the CLS assays are indicated on the left.

**b,** The actual data of colony formation units at the onset of a typical CLS aging assay for data presented in Figure **2a**. None were significant when compared with the control (0% D_2_O addition). n=3. Error bars =SEM.

**c,** The mean cell volumes of yeast grown for 2 days were determined by a Coulter counter (n=3). P values from t-tests are indicated above each bar if significant between the treatment and control (0% D_2_O addition) (< 0.01). Error bars=SEM.

**d,** Yeast growth curves under heavy water treatment in three strains (n=4). The optical density at 600nm was monitored on a Tecan plate reader for two days after inoculation. Error bars=SEM.

**e,** The quantitative characteristics of the growth curves in **d** were determined by grofit (illustrated in the first panel, n=4). P values from t-tests are indicated above each bar if significant between that treatment and control (0% D_2_O addition) (< 0.01). Error bars=SEM.

**f,** D_2_O exposure at stationary phase also extended the CLS of yeast cells (n=3). The extensions were 31% for DBY746 (P=0.02, log-rank test) and 21% for BY4743 (P=0.001, log-rank test), respectively.

**g,** Minimal effects of pre-exposure to D_2_O on growth potential in yeast. Yeast cells (BY4741) from cultures in SDC media that were previously either exposed or not exposed to D_2_O or previously exposed to were used as seeds for following growth assays with D_2_O treatments. Non-exposed: 3 days in medium with 0% D_2_O (solid lines); exposed: 40 days in medium with 50% D_2_O (dotted lines). The optical density at 600nm was monitored on a Tecan plate reader for two days after inoculation. Each line represents the growth curve of a single culture, n=6.

**h,** Growth curves of colonies (non-exposed vs. exposed, as in **G**) are overlaid for each D_2_O concentration used in the growth experiment.

**Supplementary Figure 3. Measurement of endogenous reactive oxygen species in living yeast cells. Related to Figure 3.**

The ROS production *in vivo* was monitored by fluorescence of dyes CellROX (total ROS) and MitoSOX (mitochondrial ROS) on a plate reader (n=6). Continuous yeast cultures at stationary phases were sampled at day 3, 10, and 17 after inoculation. The strains and culture conditions are indicated in the graphs. Media blank was SDC media with respective fluorescent dyes. A 2Way ANOVA test and the Area Undercurve Calculation (summarized in **Fig. 3c**) were performed in Graphpad Prism (v6.01). For CellROX fluorescence measurement, the respective P values between control and D_2_O treatment are: BY4741, <0.0001; DBY746, 0.0055; BY4743: <0.0001. For MitoSOX fluorescence measurement, the respective P values between control and D_2_O treatment are: BY4741, <0.0001; DBY746, <0.0001; BY4743: 0.0003.

**Supplementary Figure 4. The metabolome change by incorporation of deuterium through heavy water. Related to Figure 4.**

**a,** All 4,008 metabolites (scored as mass features) were examined by LC/MS and clustered from the cellular metabolome of BY4741 yeast cells upon D_2_O treatment at day 3 and day 7, using multivariate clustering model O2PLS-DA and univariate scaling (R2Y=0.993, Q2=0.979). Each circle represents one of two technical replicates each from three biological samples.

**b,** Cellular levels of monoisotopic metabolites upon D_2_O treatment. Quantitative traces of all light-isotope forms of three metabolites from yeast cells grown in control (0% D_2_O, gray) or in 50% D_2_O (red) media.

**c,** Mass spectra of tryptophan from yeast cells in **b** upon D_2_O treatment.

**d,** Medium metabolite consumption pattern altered by D_2_O treatment (as **Fig. 4d**). The medium levels of six metabolites in the first 3 days of culture after inoculation for two strains (DBY746, BY4741, n=3 each). Error bars =SEM.
