# Supplementary figures and images for: Yeast longevity promoted by reversing aging-associated decline in heavy isotope content
Source: NPJ Aging Mech Dis. 2016 Feb 18;2:16004–. doi: 10.1038/npjamd.2016.4 (PMC5515009; doi:10.1038/npjamd.2016.4)

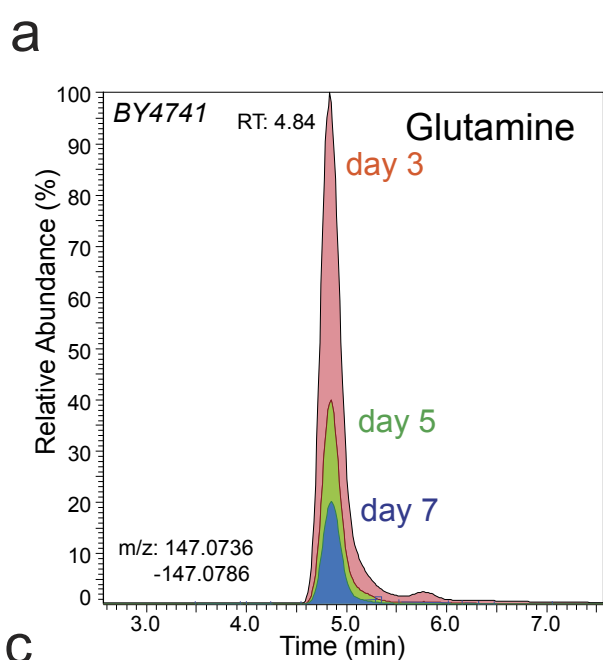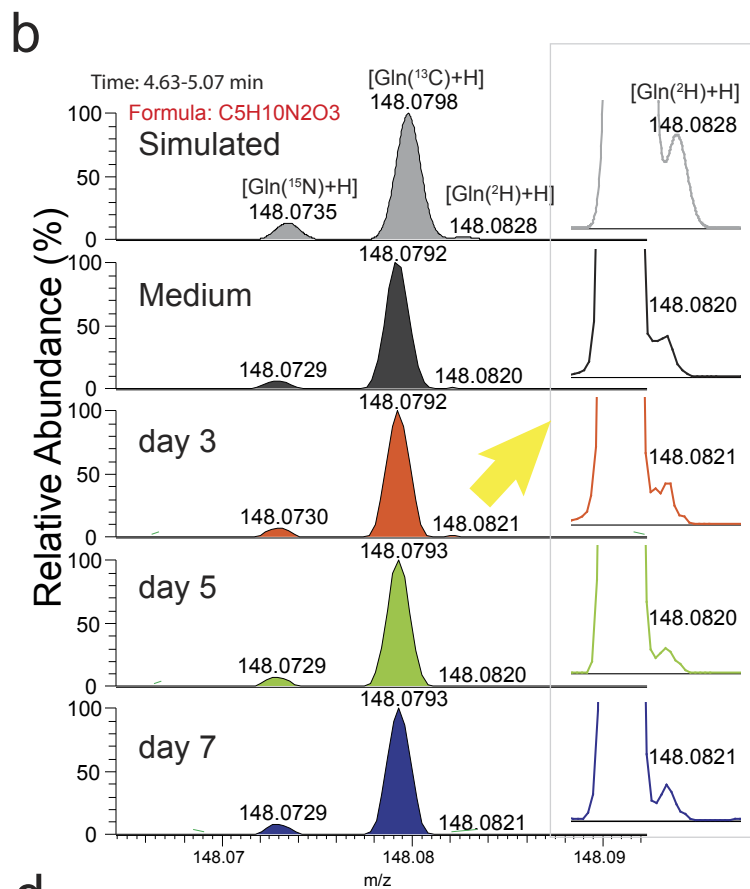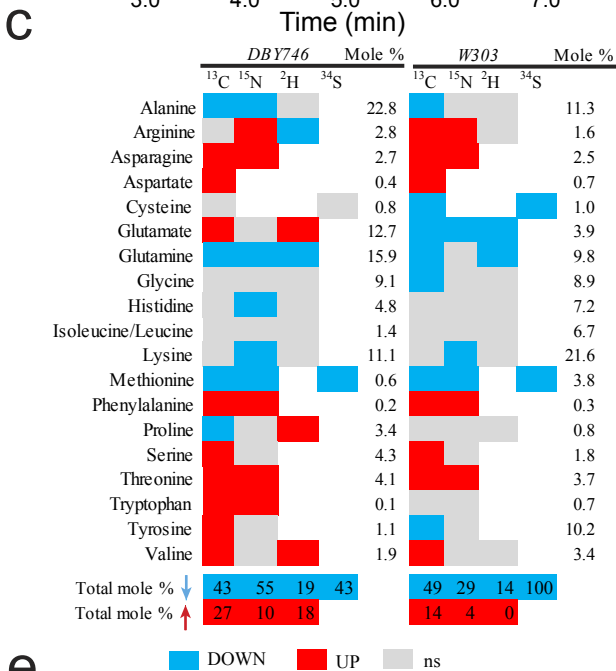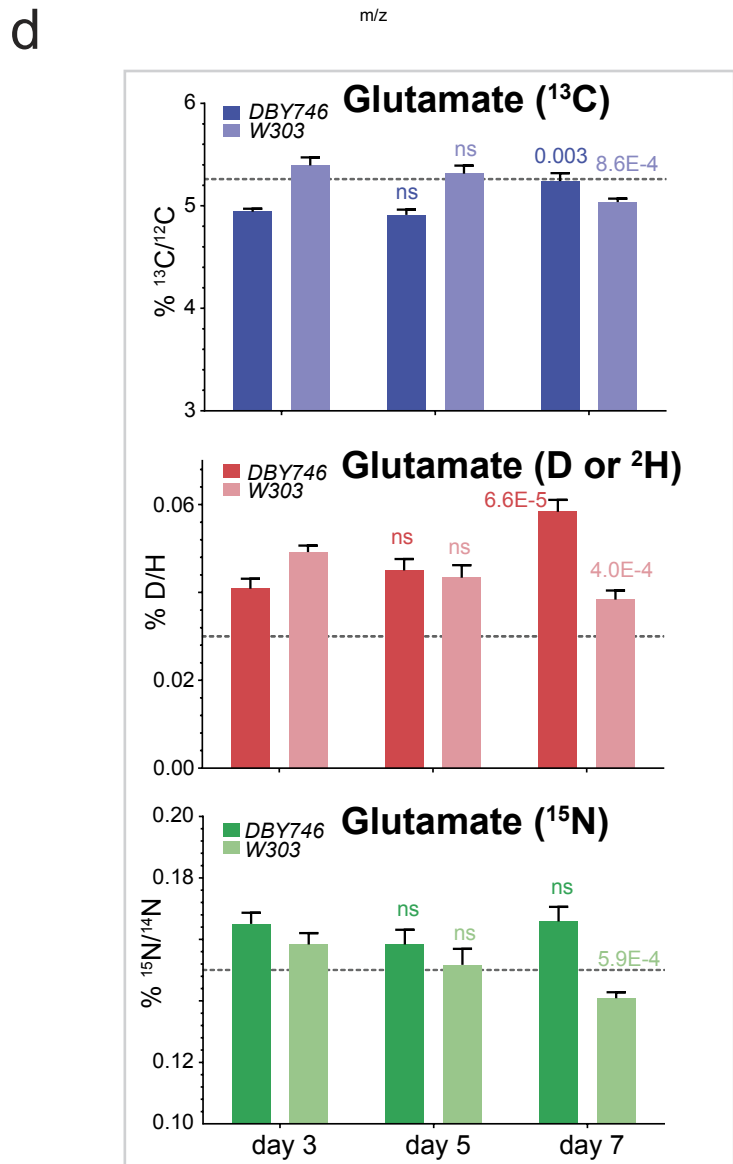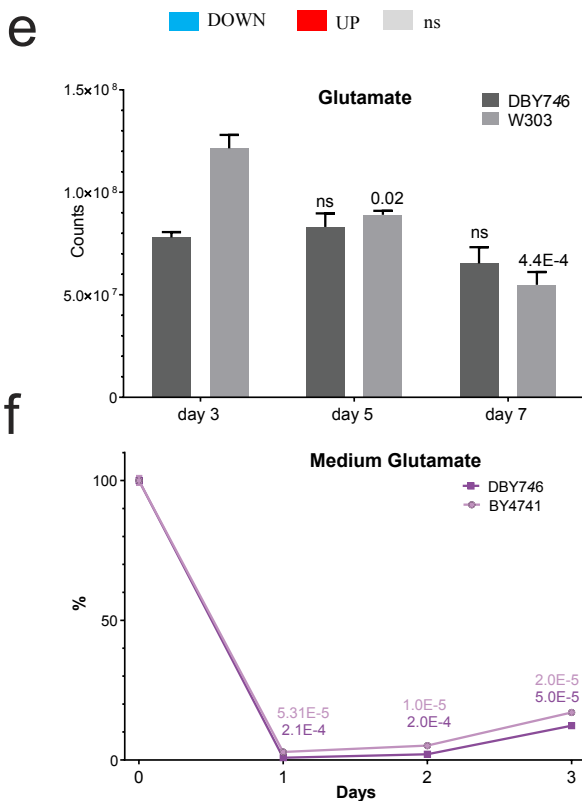

Supplement: Supplementary Figure S1 [file npjamd20164-s2.pdf]

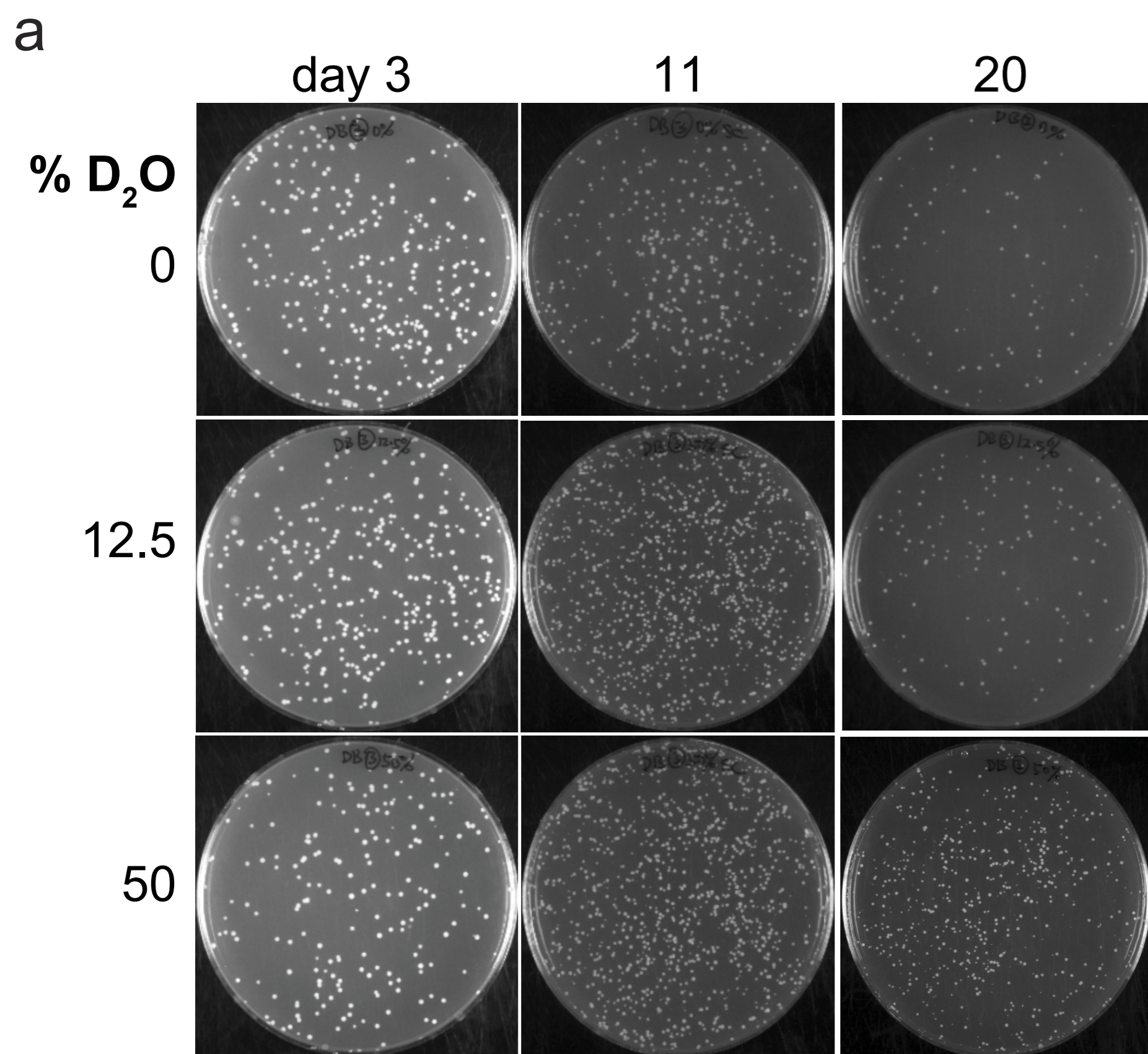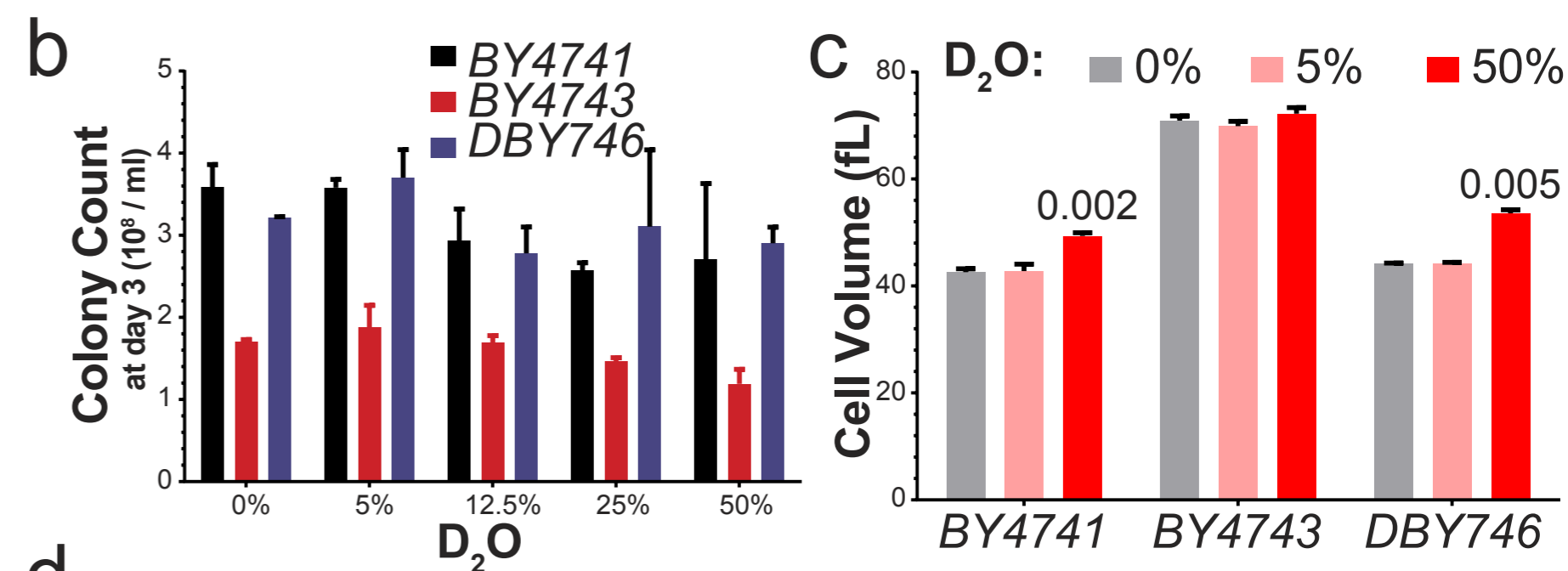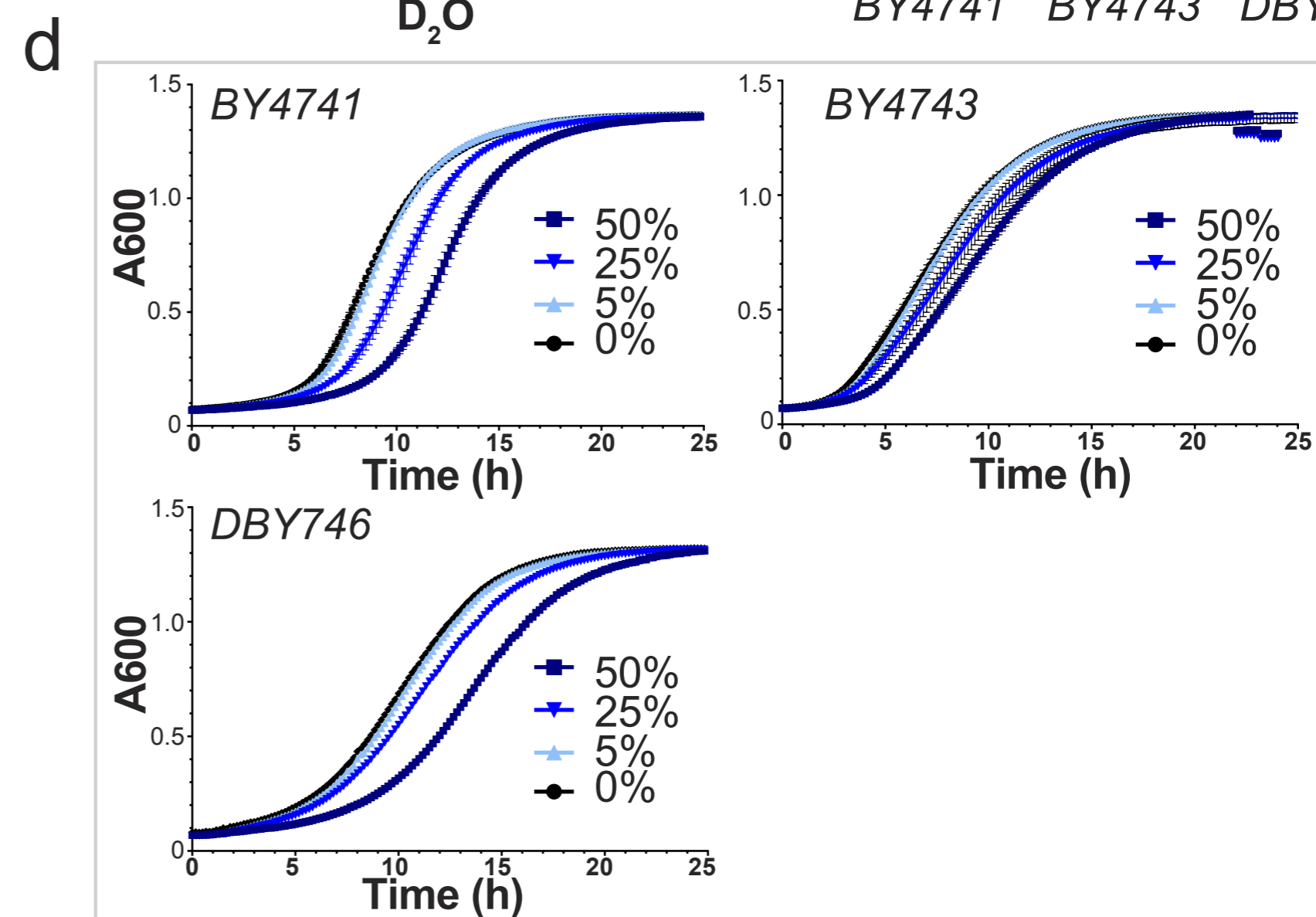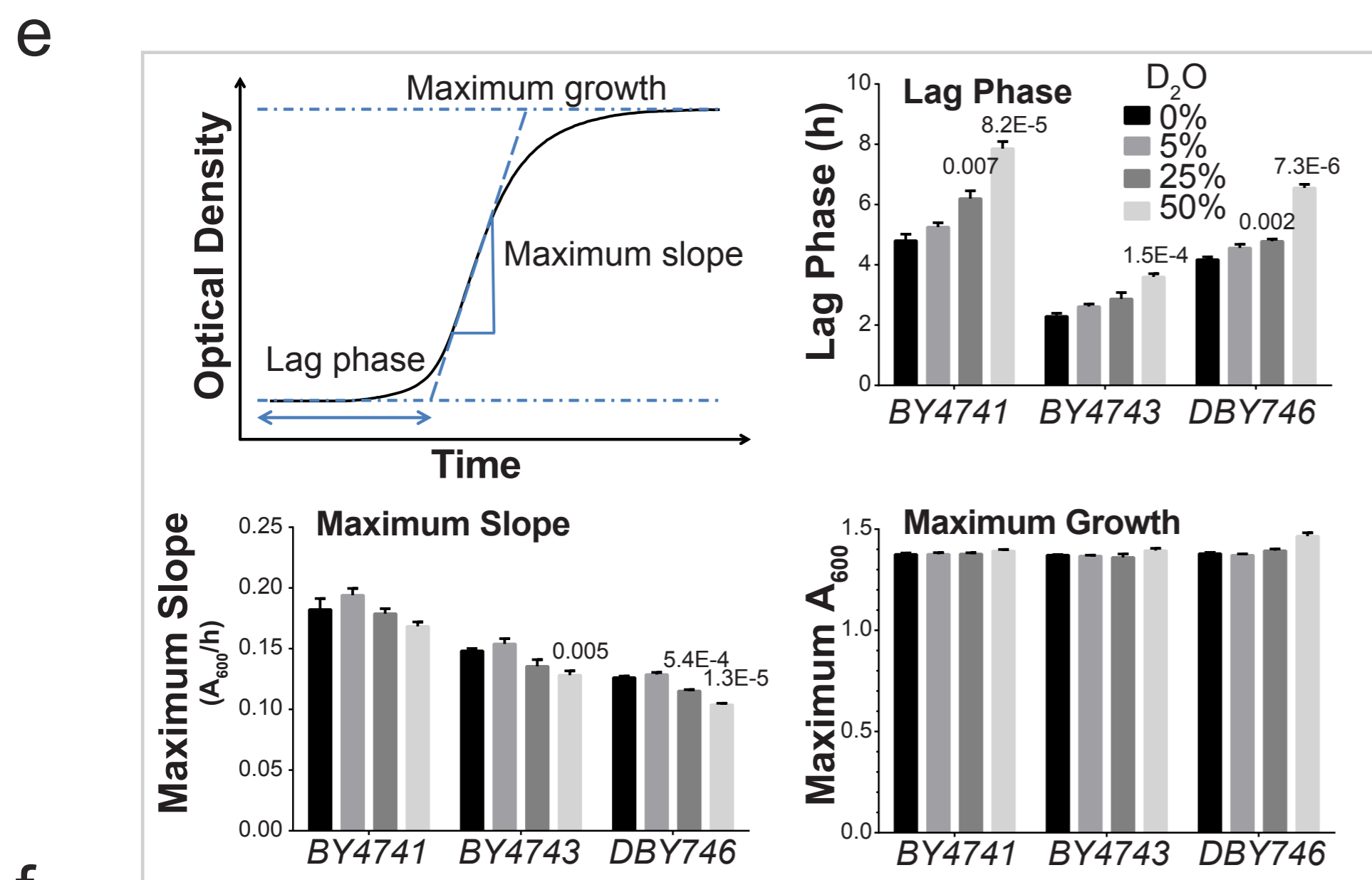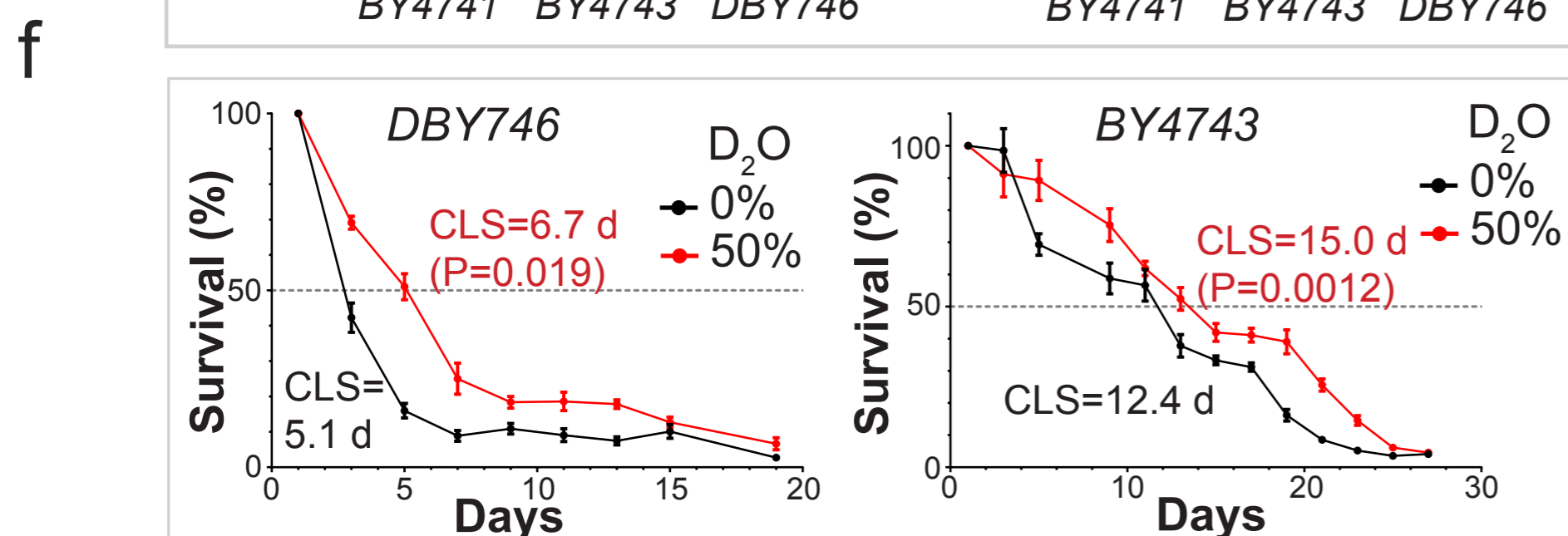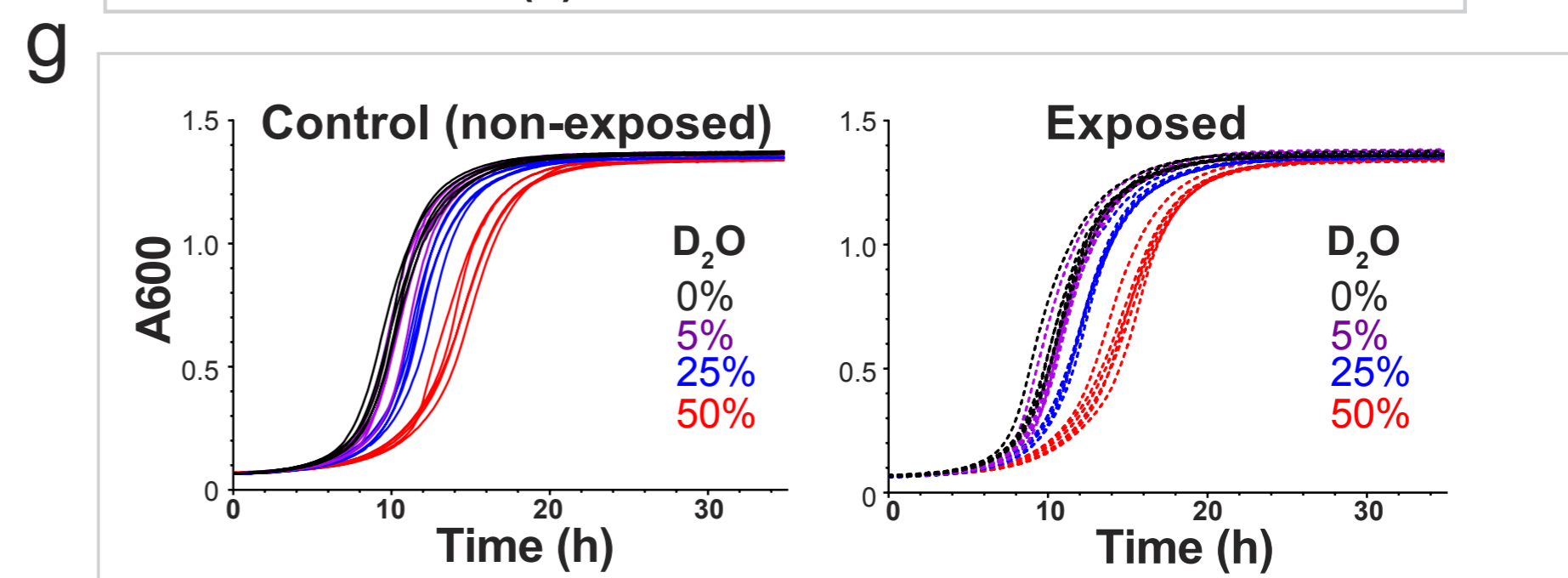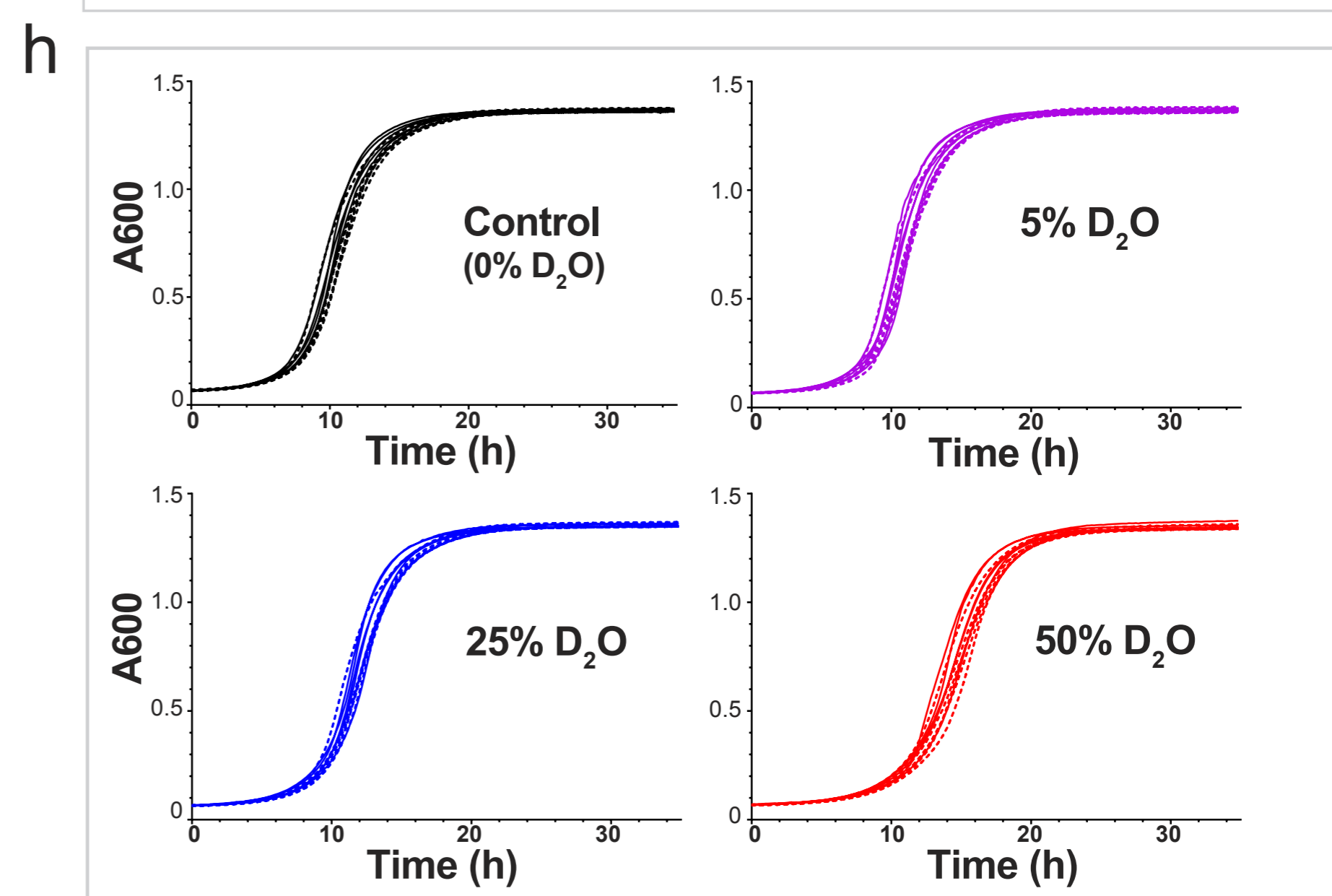

Supplement: Supplementary Figure S2 [file npjamd20164-s3.pdf]

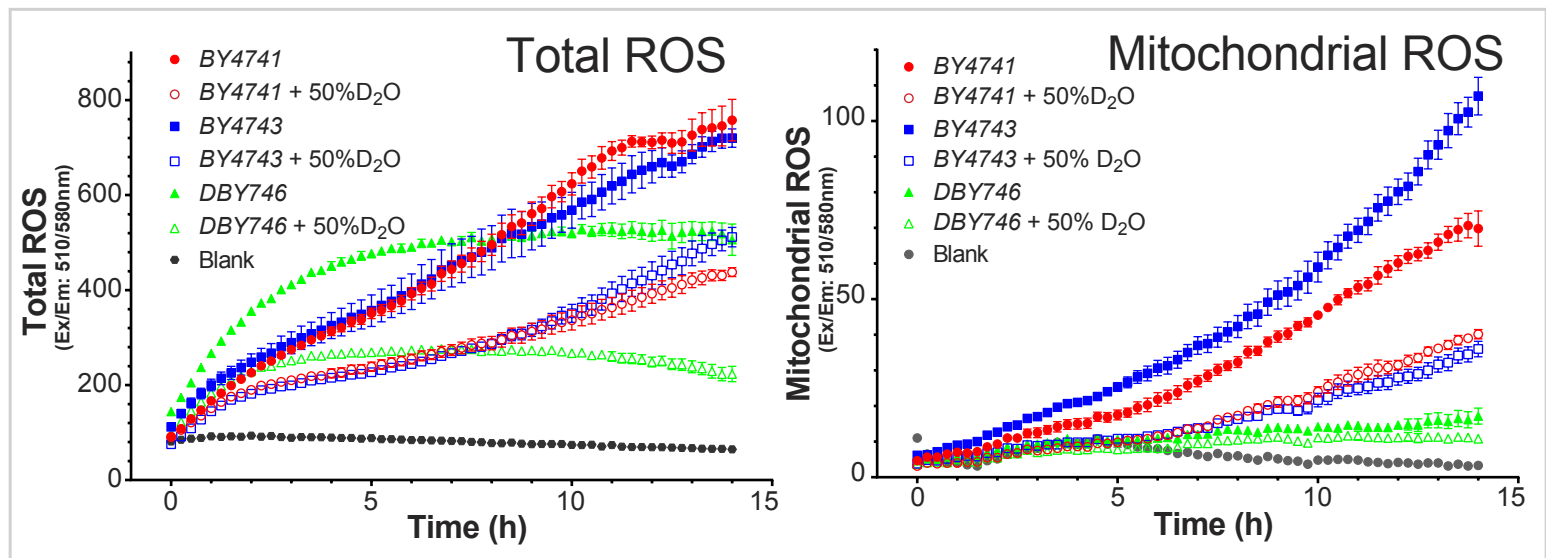

Supplement: Supplementary Figure S3 [file npjamd20164-s4.pdf]

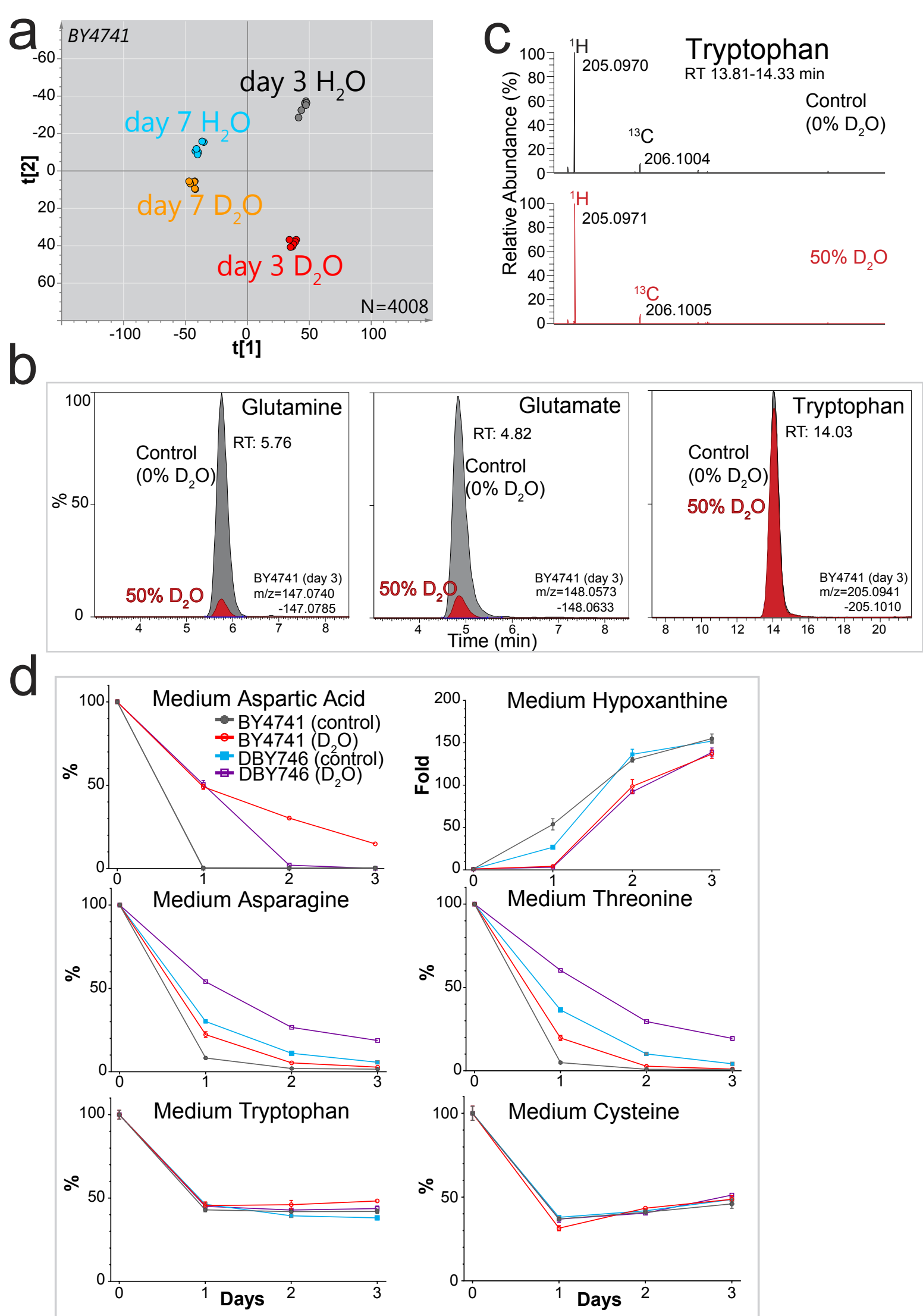

Supplement: Supplementary Figure S4 [file npjamd20164-s5.pdf]
